# Necrotizing tracheobronchitis causing airway obstruction complicated by pandemic 2009 H1N1 influenza: A case report

**DOI:** 10.1097/MD.0000000000018647

**Published:** 2020-01-03

**Authors:** Jinsun Chang, Tae-Ok Kim, Joon-Young Yoon, Bo-Gun Kho, Hong-Joon Shin, Yong-Soo Kwon, Yu-Il Kim, Sung-Chul Lim

**Affiliations:** a Department of Internal Medicine, Mokpo Hankook Hospital, Jeollanamdo; b Department of Pulmonology and Critical Care Medicine, Chonnam National University Hospital, Gwangju, South Korea.

**Keywords:** airway obstruction, influenza, necrotizing tracheobronchitis

## Abstract

**Rationale:**

Influenza is an infection caused by the influenza virus, and its symptoms are mostly mild and self-limiting. However, influenza can cause severe or fatal complications in high-risk patients. Although tracheobronchitis is one of the common complications of influenza, necrotizing tracheobronchitis is very rare. Herein, we describe a case of necrotizing tracheobronchitis causing airway obstruction complicated by pandemic 2009 H1N1 influenza.

**Patient concerns:**

A 60-year-old man presented with fever and dyspnea. On arrival at the emergency room (ER), the patient received oxygen 4 L/minute via a nasal prolong owing to mild hypoxemia. And invasive mechanical ventilation was needed 5 hours after arrival at the ER due to progressive hypoxemia.

**Diagnoses:**

Fiberoptic bronchoscopy was performed owing to bloody secretion in the endotracheal tube and revealed diffuse tracheobronchitis with necrotic and hemorrhagic materials obstructing the trachea and bronchus. The pandemic 2009 H1N1 influenza virus was detected from the bronchial washing sample; no other microorganism was detected.

**Intervention:**

He received peramivir plus oseltamivir and broad-spectrum antibiotics.

**Outcomes:**

The bloody secretion continued. He developed cardiac arrest due to airway obstruction on the 6th day of admission. After cardiac arrest, his condition progressed to multi-organ failure, and the patient died on the 10th day of admission.

**Lessons:**

We suggest that necrotizing tracheobronchitis be considered in patients with influenza who present with unexplained hypoxemia.

## 1 Introduction

Influenza, an infection caused by the influenza virus, mostly presents with mild and self-limiting symptoms. However, influenza can be accompanied by severe or fatal complications in high-risk patients, such as older patients, pregnant women, and immunocompromised patients.^[1]^ Influenza-related fatal complications include pneumonia, acute respiratory distress syndrome (ARDS), diffuse alveolar hemorrhage (DAH) syndrome, and myocarditis.^[1]^ Necrotizing tracheobronchitis is also one of the severe and fatal complications of influenza. Necrotizing tracheobronchitis complicated by influenza has been identified in autopsy studies involving pandemic 1918 H1N1 influenza patients.^[2]^ It has also been reported in autopsy studies involving pandemic 2009 H1N1 influenza patients.^[3]^ To date, few case reports have described necrotizing tracheobronchitis complicated by influenza with bacterial co-infection.^[4]^ Herein, we present a rare case of necrotizing tracheobronchitis complicated by influenza without bacterial co-infection.

## 2 Methods

This study was approved by the Institutional Review Board of the Chonnam National University Hospital (the number of approval: CNUH-EXP-2019–216). Written informed consent was obtained from the family of patient for publication of this case report and any accompanying images.

## 3 Case presentation

**Figure 1:**
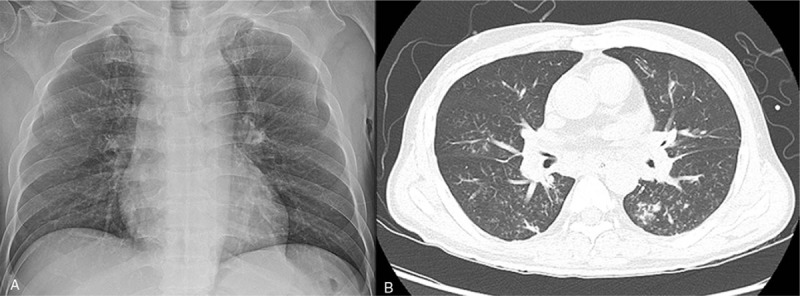
Initial chest radiograph and CT. (A) Chest radiograph at emergency room arrival. (B) Chest CT showing centrilobular nodules with linear branching opacities and confluent consolidation in both the lungs. CT = computed tomography.

**Figure 2:**
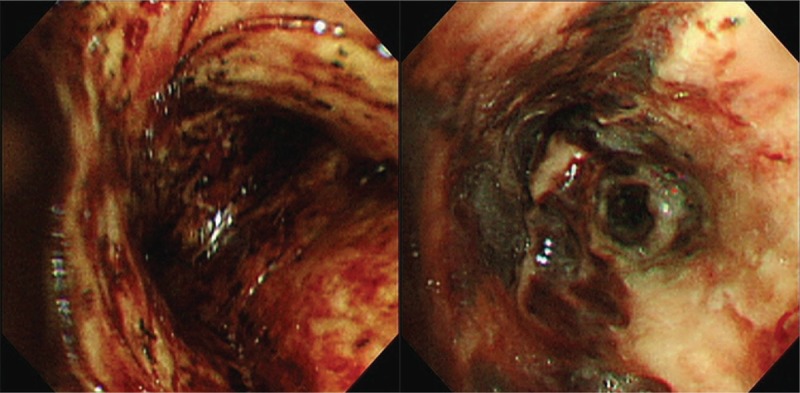
Bronchoscopy. Bronchoscopy showing necrotic mucosal changes with hemorrhagic materials and pseudomembranous changes in carina and bronchus intermedius.

**Figure 3:**
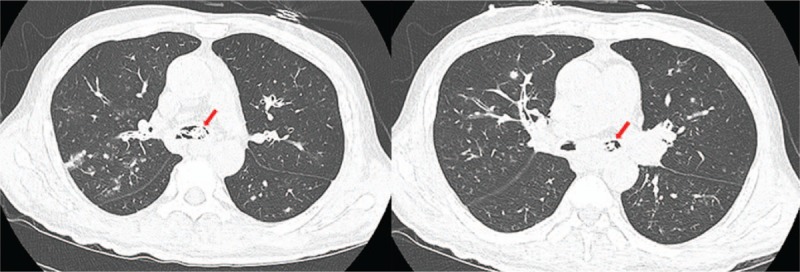
Chest radiography after cardiac arrest. Chest computed tomography showing massive materials in the trachea and both bronchi and no interval changes in centrilobular nodules with confluent consolidation in both the lungs. Arrow: endobronchial materials in both the bronchial trees.

A 60-year-old man presented to the emergency room (ER) with 2-day histories of cough, fever, dyspnea, and left hemiparesis. He had diabetes mellitus and was a current smoker. On arrival at the ER, his vital signs were as follows: blood pressure, 110/80 mmHg; respiratory rate, 22 breaths/minute; heart rate, 90 beats/minute; and oxygen saturation, 88%. On physical examination, lung sound was clear. However, neurologic examination showed grade 2/5 left hemiparesis, dysarthria, and left facial palsy. Initial chest radiography showed no pneumonic infiltrates in both the lungs (Fig. 1A). Laboratory examination performed at the ER showed the following results: white blood cell count, 22.4 × 10^3^/mm^3^; hemoglobin, 10.7 g/dL; platelet, 291 × 10^3^/mm^3^; blood urea nitrogen, 30.7 mg/dL; creatinine, 1.57 mg/dL; glucose, 305 mg/dL; C-reactive protein, 35.32 mg/dL; procalcitonin, 0.580. Liver function test and electrolyte level measurement showed normal results. Influenza A virus was detected using the rapid influenza diagnostic test (Asan easy test influenza A/B, Asan Pharm. Co., Seoul, South Korea). Brain magnetic resonance imaging showed acute infarctions scattered in the right frontal lobes, parietal lobes, and basal ganglia. At the time of ER visit, the patient received oxygen 4 L/minute via a nasal prolong. Invasive mechanical ventilation was needed 5 hours after arrival at the ER due to progressive hypoxemia. After intubation, chest computed tomography (CT) showed focal centrilobular nodules with linear branching opacities and confluent nodular consolidation in both the lungs and partially filled materials in both the bronchial lumens (Fig. 1B). The patient was admitted to the neurologic intensive care unit. We started administration of aspirin and clopidogrel for management of cerebral infarction; single-dose peramivir 600 mg and broad-spectrum antibiotics (piperacillin/tazobactam and levofloxacin) were administered intravenously. On day 2 following the ER visit, fiberoptic bronchoscopy was performed due to continuous bloody discharge and showed diffuse tracheobronchitis with necrotic and hemorrhagic materials partially obstructing the trachea and both the bronchi (Fig. 2). The pandemic 2009 H1N1 influenza virus was detected from the bronchial washing sample; however, tests for other microorganisms, such as bacteria, fungi, and *Mycobacterium tuberculosis*, were negative. Biopsy of the right main bronchus showed necrotizing inflammation. After bronchoscopy, he received oseltamivir 75 mg twice a day for extended therapy. However, we observed continuous bloody discharge through the endotracheal tube. On day 6, cardiac arrest occurred due to airway obstruction. After 6 cycles of cardiopulmonary resuscitation, spontaneous circulation returned. After the patient recovered from cardiac arrest, CT showed a large amount of materials in the endo-tracheobronchial lumen and no changes in infiltration in both the lungs (Fig. 3). After cardiac arrest, the mechanical ventilator showed high peak pressure (50–55 mmH_2_O) and low plateau pressure (14 mmH_2_O). On day 10, he died due to progression to multiple organ failure after cardiac arrest.

## 4 Discussion and conclusions

Influenza, an infection caused by the influenza virus, is mainly prevalent in the winter season. Although its symptoms are mostly mild and self-limiting, influenza can lead to life threatening conditions, such as necrotizing tracheobronchitis, pneumonia, ARDS, and DAH syndrome. Pneumonia and ARDS were the most common causes of death in patients with pandemic 2009 H1N1 influenza.^[5]^

Necrotizing tracheobronchitis caused by influenza has been mostly reported in autopsy studies. It was first reported in autopsy studies involving pandemic 1918 H1N1 influenza patients. In these autopsy studies, histologic findings included diffusely swollen and inflamed bronchial surfaces, evidence of hemorrhagic bronchitis and tracheobronchitis, and luminal filling with frothy blood-stained material.^[2]^ This disease related to pandemic 2009 H1N1 influenza was also reported in autopsy studies. Histopathological examination of the tracheal and bronchial specimens showed mucosal/submucosal mononuclear cell infiltration, multifocal desquamation of the epithelium, congestion and hemorrhage, and necrotizing tracheobronchitis.^[3]^

Necrotizing tracheobronchitis is very rare, and it can be caused by viral and bacterial infections, mechanical ventilation, rheumatoid arthritis, and ulcerative colitis.^[6–8]^ All these diseases were confirmed by histologic examination through bronchoscopy. Necrotizing tracheobronchitis is treated with the aim of controlling the underlying disease.

Bacterial co-infection is one of the common causes of influenza-related deaths. Co-infection of the pandemic 2009 H1N1 influenza virus and bacteria has been reported in 22% to 30% of critically ill patients with influenza.^[9,10]^ Recently, necrotizing tracheobronchitis due to co-infection of the pandemic 2009 H1N1 influenza virus and methicillin-resistant *Staphylococcus aureus* (MRSA) has been reported.^[4]^ Unlike this previous case, there was no evidence of bacterial co-infection in our case. Consistent with our findings, an autopsy study reported that pandemic 2009 H1N1 influenza can cause necrotizing tracheobronchitis without bacterial co-infection.^[3]^

In conclusion, necrotizing tracheobronchitis can be caused by influenza alone without bacterial co-infection. Necrotizing tracheobronchitis should be considered in influenza patients with unexplained hypoxemia. Fiberoptic bronchoscopy or CT may be considered for detecting the cause of hypoxemia.

## Author contributions

**Conceptualization:** Tae-Ok Kim.

**Data curation:** Jinsun Chang, Tae-Ok Kim, Joon-Young Yoon, Bo-Gun Kho, Hong-Joon Shin, Yong-Soo Kwon.

**Formal analysis:** Jinsun Chang, Tae-Ok Kim.

**Funding acquisition:** Tae-Ok Kim, Yong-Soo Kwon, Sung-Chul Lim.

**Resources:** Jinsun Chang, Tae-Ok Kim.

**Supervision:** Hong-Joon Shin, Yong-Soo Kwon, Yu-Il Kim, Sung-Chul Lim.

**Validation:** Tae-Ok Kim.

**Writing – original draft:** Jinsun Chang.

**Writing – review & editing:** Tae-Ok Kim.

Tae-Ok Kim orcid: 0000-0002-0922-9472.
